# HIV-1 Nef Down-Modulates C-C and C-X-C Chemokine Receptors via Ubiquitin and Ubiquitin-Independent Mechanism

**DOI:** 10.1371/journal.pone.0086998

**Published:** 2014-01-29

**Authors:** Prabha Chandrasekaran, Victoria Moore, Monica Buckley, Joshua Spurrier, John H. Kehrl, Sundararajan Venkatesan

**Affiliations:** 1 Laboratory of Molecular Immunology, National Institute of Allergy and Infectious Diseases, National Institutes of Health, Bethesda, Maryland, United States of America; 2 Laboratory of Immunoregulation, National Institute of Allergy and Infectious Diseases, National Institutes of Health, Bethesda, Maryland, United States of America; Helmholtz Zentrum Muenchen - German Research Center for Environmental Health, Germany

## Abstract

Human and Simian Immunodeficiency virus (HIV-1, HIV-2, and SIV) encode an accessory protein, Nef, which is a pathogenesis and virulence factor. Nef is a multivalent adapter that dysregulates the trafficking of many immune cell receptors, including chemokine receptors (CKRs). Physiological endocytic itinerary of agonist occupied CXCR4 involves ubiquitinylation of the phosphorylated receptor at three critical lysine residues and dynamin-dependent trafficking through the ESCRT pathway into lysosomes for degradation. Likewise, Nef induced CXCR4 degradation was critically dependent on the three lysines in the C-terminal -SSLKILSKGK- motif. Nef directly recruits the HECT domain E3 ligases AIP4 or NEDD4 to CXCR4 in the resting state. This mechanism was confirmed by ternary interactions of Nef, CXCR4 and AIP4 or NEDD4; by reversal of Nef effect by expression of catalytically inactive AIP4-C830A mutant; and siRNA knockdown of AIP4, NEDD4 or some ESCRT-0 adapters. However, ubiquitinylation dependent lysosomal degradation was not the only mechanism by which Nef downregulated CKRs. Agonist and Nef mediated CXCR2 (and CXCR1) degradation was ubiquitinylation independent. Nef also profoundly downregulated the naturally truncated CXCR4 associated with WHIM syndrome and engineered variants of CXCR4 that resist CXCL12 induced internalization via an ubiquitinylation independent mechanism.

## Introduction

The Human Immunodeficiency Virus (HIV) encoded membrane-associated myristoylated Nef protein is a virulence factor critical for enhanced viral replication, immunopathogenesis and immune evasion [1], [2], [3]. Nef is a multivalent adaptor protein, that modulates the trafficking and signaling of numerous immune cell receptors including CD3,CD4, CD8,MHC class I (MHC-I), CD1a, CD1d, the invariant chain of immature MHC-II (CD74), mature MHC-II, DC-SIGN, mannose receptor, tumor necrosis factor, CD80, CD86, transferrin receptor, CTLA-4 and hemochromatosis protein HFE [4], [5], [6], [7], [8], [9], [10], [11], [12], [13], [14], [15], [16], [17], [18]. More recent reports have shown that different chemokine receptors (CKRs) including the HIV co-receptors CCR5 and CXCR4 [19], [20], [21] are also downregulated by Nef. Through its effects on CD4 and HIV co-receptors, Nef induces super-infection immunity [21] and enhanced virus replication [22]. However, relative to CD4 and MHC-I, there is a limited knowledge of the mechanism(s) or the functional consequence of chemokine receptor modulation.

T lymphocytes employ several different CKRs and their ligands to regulate T-cell ontogeny in the thymus [23], [24], [25] and adaptive immune response in the periphery [26], [27]. Correspondingly, chemokine and chemokine receptor dysfunction is associated with numerous acute and chronic immune diseases such as asthma and arthritis and infectious diseases. Among the many CKRs, CXCR4 has been identified for a previously unrecognized role as a costimulator that functions together with the pre-TCR (T-cell receptor) to promote the DN3-to-DN4 (double negative 3 to double negative 4) transition in the thymus [25]. Transgenic mice expressing HIV-1 provirus or the viral Nef protein alone reproduce the general pathology and immune dysfunction of AIDS [1], [28] and display progressive depletion of double positive cells and a CD4^+^ single positive T cell generation defect in the thymus [29]. Taken together, these observations suggest that Nef induced defects in T cell development reflect reduced CXCR4 levels and/or inadequate CXCR4 signaling.

Signal transduction through CKRs is modulated by three distinct mechanisms: desensitization, internalization and recovery. Receptor desensitization occurs rapidly after agonist binding and is mediated by phosphorylation of the receptor by G protein-coupled receptor kinase(s) (GRKs) followed by recruitment of β-arrestin, which uncouples the CKRs from G protein activation [30], [31] and facilitates recruitment of the CKRs into endocytic vesicles through interactions of β-arrestin with clathrin and the β2 subunit of the AP2 adapter complex [32], [33]. Internalized receptors are either recycled back to the PM or sorted to lysosomes for proteolysis. Different mechanisms control GPCR (G-protein coupled receptors) trafficking itineraries [34]. Even at the first stage of endocytic sorting at the plasma membrane, GPCRs can differ as to whether they are recruited into clathrin-coated pits, or not. Once delivered to early endosomes receptors are sorted between lysosomal and recycling pathways [35], [36]. Lysosomal sorting is the preferred itinerary for many GPCRs and receptor tyrosine kinases that are downregulated by ligand binding [37], [38], while GPCRs sorted to recycle may rapidly reappear at the plasma membrane thereby restoring cell signaling [39], [40], others continue to signal from the endosomal membrane [41].

Some receptors such as CXCR4 and β-AR (β-adrenergic receptors) are sorted to lysosomes through the ESCRT (Endosomal Sorting Complex Required for Transport) machinery after ubiquitinylation following agonist binding [42], [43]. In the case of CXCR4, ubiquitinylation of agonist occupied CXCR4 is mediated by AIP4 (Atropin Interacting Protein 4), a HECT (homologous to E6-AP carboxy terminus) domain E3 ubiqutin ligase [44]. Three lysine residues in a degradation motif near the C-terminus of CXCR4 were required for ubiquitinylation. Here we show that Nef acts as an intracellular surrogate agonist for CXCR4 by directly recruiting the HECT domain E3 ligases to ubiquitinylate CXCR4. This led to dynamin dependent endocytosis into MVBs (multi-vescicular bodies) for proteolysis. Following agonist binding or Nef expression, other CKRs like CXCR2 were also targeted for degradation, but in ubiquitinylation independent manner.

## Experimental Procedures

### Expression plasmids, siRNAs, Antibodies and Reagents

Expression plasmids for wild type (wt) and mutant CKRs and plasmids for CXCR4 or CCR5 C-terminally tagged with 6-HIS (6× Histidine) or FLAG have been described before [45], [46], [47], [48]. Joshua Farber (LMI, NIAID) provided expression plasmids for CCR7 and CXCR5, while the natural WHIM (WM, mutation associated with WHIM (Warts, hypogammaglobulinemia, infections, and myelokathexis) syndrome) CXCR4 mutant truncated at residue 334 and CHO and K562 cell lines expressing wt or WM CXCR4 were obtained from David McDermott (LMI, NIAID NIH). Expression plasmids for HA (hemagglutinin) tagged wt, phosphorylation deficient (S3245A) and LYS/ARG (K327/331/333R) CXCR4 mutants were obtained from Adriano Marchese (Stritch School of Medicine, Loyola University, Chicago) as were the plasmids expressing wt AIP4 or NEDD4 and c-myc or FLAG tagged wt AIP4 and c-myc tagged Q297A/N329A mutant that does not bind to CXCR4 and C830A AIP4 mutant lacking E3 ligase activity. YFP (yellow fluorescent protein) tagged CXCR4 was engineered by transferring CXCR4 ORF upstream of YFP ORF in p-EYFP-N1 plasmid (Clontech Corp). The wt, null (NefXho) or indicated mutants in the NL4-3 or NA7 Nef backbone and various HIV or SIV Nef alleles had been cloned in the pCG vector with a 3′HA or 6-His, Cerulean (Cer) or Green Fluorescent Protein (GFP) tags as described before [49]. Nef and a null mutant, NefXho [50] were also cloned in a bicistronic pIRES vector (Clontech, Mountain View, CA) upstream of GFP ORF. The HIV proviral DNAs expressing murine HSA (head stable antigen, CD24) and VSV-G (Vesicular Stomatitis Virus) DNA were obtained from NIH AIDS Reagent and Reference Program. The initiator MET codon of Nef was mutated in this construct [49] and used as the Nef-deficient virus in this study. RNA-mediated interference of ß-arrestin and clathrin heavy chain was performed using small interfering RNA duplexes (siRNAs) of the following sequence: ß-arrestin 2&3, ACCUGCGCCUUCCGCUAUGUU; clathrin heavy chain (CHC), CCUGCGGUCUGGAGUCAACUU (Dharmacon RNA Technologies, CO). The siRNA sequences designed for the knock down of AIP4 and NEDD4 were UUUCAAUGCAGAAUUUCUGUGGUCC and UAGAGGAGAAGGUUCUUGUUGUUGC, respectively (Invitrogen, CA). The AP1γ chain, AP2α chain, Hrs/Vps27 and USP14 were knocked down using the respective on-target Smart Pool siRNAs from Dharmacon.

Antibodies against β-arrestin, clathrin, Hrs/Vps27, USP-14 and HA epitope were purchased from Abcam, MA; rat antibody against HA was obtained from Roche Inc., antibodies against actin, AIP4 and NEDD4 from Santa Cruz, CA; anti-FLAG M2 antibody and conjugated beads from Sigma Aldrich (St. Louis, MO); antibodies against AP2α1 and AP1γ from Affinity Bioreagents; and unconjugated or Alexa dye conjugated antibodies against CD4, CCR2A/2B, CCR3, CCR5, CCR7, CXCR-1, 2, 3 & 4, anti-mouse CD24 were purchased from R&D systems, MN or BD Biosciences, CA. Nickel NTA agarose used for immuno-precipitation was from Qiagen, CA, and the histidine-tagged proteins were detected in the blot using HisProbe HRP conjugate (Thermo Scientific, Rockford, IL). Alexa dye conjugated secondary antibodies were from Invitrogen, CA and HRP conjugated goat anti-murine, rabbit or human IgG and donkey anti-goat IgG were from Pierce Corp. The drugs were purchased from the following sources: Dyansore from Tocris, MO, Epoxomicin and lactacystin from EMD chemicals, NJ. CXCL12 and RANTES were from R&D systems and respectively.

### Cells, transfections and siRNA knockdowns

Human T cell lines used in this study are CD4^+^Jurkat and CEM cells as reported before [51]. Hela cells were used for microscopy and T cell lines of Jurkat or CEM cells were used for phenotypic assays. Hela cell transfection conditions have been described in detail before [51]. The Department of Transfusion Medicine at NIH provided elutriated monocytes and leukocyte enriched buffy coat from anonymous volunteers. PBLs were purified as before and cells were cultivated under standard conditions in RPMI or DMEM medium with 10% fetal calf serum (FCS) and 1% L-glutamine, as appropriate. The cells were cultivated under standard conditions in RPMI or DMEM medium with 10% fetal calf serum (FCS) and 1% L-glutamine, as appropriate. K562 cell lines, Hela cells and Chinese hamster ovary (CHO) cells used for transfections with plasmids of CKRs and their mutants were obtained from ATCC (Rockville, MD). CEM.NKR-CCR5 cell line generated by Dr. Alexandra Trkola [51] was obtained through the NIH AIDS Reagent Program, Division of AIDS, NIAID, NIH.

To determine the downregulation levels of various CKRs, nucleofection of cell lines, PBLs or monocytes was carried out with 3–5 µg of plasmids at a cell density of 5–7×10^6^ cells/100 µl of Nucleofector solution with the Amaxa Nucleofector device using programs recommended by the manufacturer (Lonza, Germany). The plasmids encoding eGFP was co-transfected to monitor the transfected cells by flow cytometry. siRNA knockdowns were performed in the Jurkat cells using Amaxa nucleofector device (Lonza) as described before [50]. For confocal microscopic experiments, Hela cells were transfected with plasmids, using oligofectamine (Invitrogen).

To assess the levels of CKRs modulated by Nef, HEK cells were transfected with plasmid encoding Nef, CD8 alpha chain as a transfection marker and HA tagged CXCR4, or its mutants, WHIM CXCR4/CXCR1/CXCR2. For experiments assessing the role of AIP4 in the down regulation of CKRs, myc tagged wt/C830A AIP4 were also co-transfected with the above-mentioned plasmids.

### Flow cytometric analysis

Cells were stained with the fluorochrome conjugated antibodies against CKRs and transfection markers in 1XPBS containing 1% goat serum for 15 min at room temperature. The cells infected with HIV-1 viruses were stained for mouse CD24 in combination with the other markers to be able to gate for the infected cells during acquisition and analyses. The data acquisition was carried out using a two laser, four channel FACSort™ (BD biosciences) flow cytometer and the analyses were done using FlowJo version 7.1.3. (Tree Star Inc., Ashland, OR).

### Drug treatments and Endocytosis assay

The drug treatments and the endocytosis assay were performed in Jurkat cells expressing either pCG vector or pCG-Nef and GFP. For both assays the cells were washed with HBSS (Gibco, Invitrogen). Drug treatments were done on cells suspended in HBSS for 4 h for Dynasore (80 µM) and 5 h for lactacystin and Epoxomicin (25 µM) at 37°C. The cells were then collected for flow cytometric analyses. For agonist dose response and endocytosis rate assays, the cells were treated with the respective agonists for CKRs as described in detail before [48].

### Immuno-precipitation

For immuno-precipitation studies lysates were used either from untransfected cells or cells transfected with plasmids encoding Nef, CXCR4-His and FLAG-wt/mutant AIP4. The cell pellets were suspended in the lysis buffer (20 mM Tris pH 8.0, 150 mM NaCl, 1% Triton X-100, and protease inhibitor mix (Roche Molecular Biochemicals) and disrupted by three cycles of freeze-thawing. The lysates were incubated with Ni-NTA agarose for 6 hours at 4°C, washed and eluted by boiling in LDS sample buffer (Invitrogen). The co-precipitated AIP4 was detected using anti-Flag monoclonal antibody (M2 Flag antibody, Sigma). All the proteins were resolved in NuPAGE 4–12% Bis-Tris gel (Invitrogen) and the blots were visualized using chemiluminescence detection solution (Pierce).

### Confocal Microscopy

Hela cells (0.2–0.4×10^5^) were seeded onto the cover slips in a 24 well plate, The following day the cells were co-transfected with plasmids encoding CXCR4-YFP, Nef-Cer or Cer using lipofectamine 2000 (Invitrogen). After 16 h transfection, the cells were fixed and stained for the cellular organelles using the respective murine monoclonal or rabbit polyclonal antisera, followed by a secondary staining with Alexa-647 conjugated secondary antibodies. Confocal microscopy using a Leica TCS-NT/SP5 microscope (Leica, Exton, PA USA) and image analysis as described before [49].

### Ubiquitinylation assays

Ubiquitinylation of CXCR4 was evaluated by two different methods. Jurkat cells co- transfected with GFP and Nef or null plasmids were evaluated by flow cytometry for Nef induced CD4 downregulation. After adjusting to constant levels of GFP expression, cells were disrupted and ubiquitinylated proteins were recovered using UbiQapture™-Q Kit, containing a high-binding affinity matrix of immobilized monoclonal anti-ubiquitin antibodies (ENZO Life Sciences, Germany), following manufacturer's instructions. Ubiquitinylated proteins were resolved by SDS/PAGE and CXCR4 was detected by immuno-blotting using a monoclonal antibody (Abcam). Alternatively, Nef (+) or Nef (−) transfectants co-expressing FLAG-tagged ubiquitin and HA tagged CXCR4 were lysed using the lysis buffer (150 mM NaCl, 50 mM Tris, 1% NP40, and protease inhibitor cocktail complete (Roche Diagnostics)) followed by immuno-precipitation of ubiquitinated proteins using M2 FLAG mAb agarose (Sigma Aldrich, St. Louis, MO) and immuno-blotting with anti-HA (Roche Diagnostics) to detect the ubiquitinylated receptor.

### Statistical Analysis

Statistical analyses were done using Graphpad prism version 5.0. Paired or unpaired t-tests were done as appropriate, to estimate the statistical significances observed between the means. A value of p<0.05 was considered to be significant in all the analyses. The graphs were generated using either Microsoft Excel or Graphpad prism. Data are presented as Mean+standard deviation (SD).

## Results

### Nef downregulated many chemokine receptors to variable extent in different cells

Extending the results obtained by Michel et al [21], we evaluated Nef effect on 11 different CKRs in their natural context in fresh PBMCs (peripheral blood mononuclear cells), monocytes and T cell lines, and recombinant CKRs in cell lines or transient transfectants. In the presence of Nef, the expression of surface chemokine receptors ranged from as high as 85% for CCR9 in Jurkat cells to as low as 68% for CCR3 in K562 cells indicating the variable effect of Nef on CKR expression. The downregulation of CKRs was modest when compared to that of CD4 in the same cells, which reached a maximum of two to three-fold lower levels of Nef (Table 1). Among these, CXCR4 downmodulation by Nef varied from 40% in PBLs to no downmodulation in K562 cells (Table 1). Nef effect on CXCR4 was recapitulated in T cell lines and monocytes transduced with bacterially expressed hexa-His-tagged myristoylated Nef containing TAT-Arginine-rich motifs (RRMs) (Figure S1 A) and in single cycle infection of CEM cells with VSV-G pseudotyped Nef (+) and Nef (−) HIV (Figure S1 B).

**Table 1:** HIV-1 Nef induced variable levels of chemokine receptor downregulation in different cell types.

Receptors		PBLs	Monos	Jurkat	CEM**	CHO	K562#
Examined*							
**CCR1 &**	**CCR1**			39±3		43±6	41±6
**CD4**	**CD4**			11±1		14±1	21±1
**CCR2A &**	**CCR2A**			53±3			
**CD4**	**CD4**			9±1			
**CCR2B &**	**CCR2B**	78±8$	66±7	32±4		47±4	54±6
**CD4**	**CD4**	15±1	19±2	11±1		16±1	18±1
**CCR3 &**	**CCR3**			51±6		52±7	32±4
**CD4**	**CD4**			14±1		12±1	16±1
**CCR5 &**	**CCR5**	66±5	73±6	72±5	61±0.5	67±6	71±7$
**CD4**	**CD4**	17±1	24±2	14±1	8±5	12±1	15±1
**CCR7 &**	**CCR7**	56±6		69±6			
**CD4**	**CD4**	11±1		12±1			
**CCR9 &**	**CCR9**			81±6$			
**CD4**	**CD4**			10±1			
**CXCR1 &**	**CXCR1**			47±3		41±3	82±6$
**CD4**	**CD4**			14±1			22±1
**CXCR2 &**	**CXCR2**			50±4		44±5	49±5
**CD4**	**CD4**			11±1			17±1
**CXCR3 &**	**CXCR3**			71±5			
**CD4**	**CD4**			10±1			
**CXCR4 &**	**CXCR4**	59±5	57±6	61±4	58±5	78±6$	98±6$
**CD4**	**CD4**	11±1	22±1.2	10±1	12±1	16±1	13±1
**CXCR5 &**	**CXCR5**			51±4		33±6	57±6
**CD4**	**CD4**			13±1		18±1	20±1

* For each type of cell, Nef effect on the expression of the indicated CKR was evaluated along with that of CD4 (endogenous or plasmid expressed).

** CEM-NKR cell line expressing CCR5 and CXCR4 was used.

# CXCR4 was analyzed in a K562 CXCR4 cell line.

Cells were transfected with IRES plasmid encoding Nef and or NefXho (null) and GFP. Cell surface expression of endogenous CCR2B, CCR5 (fresh PBLs, monocytes), CCR7 (PBMCs only), CXCR4 (fresh PBLs, monocytes, Jurkat and CEM cells), or from expression plasmids for the indicated CKRs (CHO or K562 or Jurkat) was evaluated by flow cytometry of GFP gated cells. MFV for GFP (+) and NefXho (null) population was set to 100 in each case. The downregulation was significant when compared to endogenous or plasmid expressed receptor levels for all the CKRs and CD4 (p<0.05), except for those denoted by ^$^.

The data in the table represents mean ± standard deviation. (n = 4, for PBMCs, monocytes, CHO and K562 cells; and n = 3 for CEM and n = 4 for Jurkat cells).

### Genetic Analysis of Nef motifs required for modulation of chemokine receptors

Nef has distinct sequence determinants for CD4 or HLA-I downregulation. For instance, N-terminal α-helix with conserved Met at position 20 (Met-20), polyproline motif ^72^PVT(R)PQVP^78^, and an acidic domain, ^62^EEEE^65^ are required for HLA-I downregulation [49], [52], [53], [54], but not for CD4 loss. Instead, alanine substitutions at the dileucine motif at 168 [Nef-LLAA] drastically reduced Nef effect on CD4 [50], [55]. We evaluated these mutants in the context of NL4-3 and NA7 Nef alleles for their effect on CKRs. In essential agreement with earlier report [24], alanine substitution mutants at the tetra-glutamate motif at 62, or at the PXXP motif were devoid of CXCR4 downregulation, while retaining the CD4 effect in Jurkat and CEM cells and PBMCs. (Figure S2 A and B). Other reports have suggested that PRO at 78 is the critical residue for HLA-I downregulation and SH3 domain binding [56], [57], [58], [59], [60]. However, our PRO mutants that were defective for HLA-I and CXCR4 downregulation changed both PROs to ALAs at the ^72^PVT(R)P^75^ or ^75^PQVP^78^ sequence and as such could not distinguish the relative contribution of individual PROs within the ^72^PVT(R)PQVP^78^ stretch [50]. Other receptors such as CCR5 in CEM cells (Figure S2 B), CXCR1 and CXCR2 (data not shown) in Jurkat transfectants displayed similar sensitivity profiles towards Nef mutants.

### Nef inhibited agonist mediated chemokine receptor internalization and did not significantly enhance their intrinsic endocytosis

**Figure 1:**
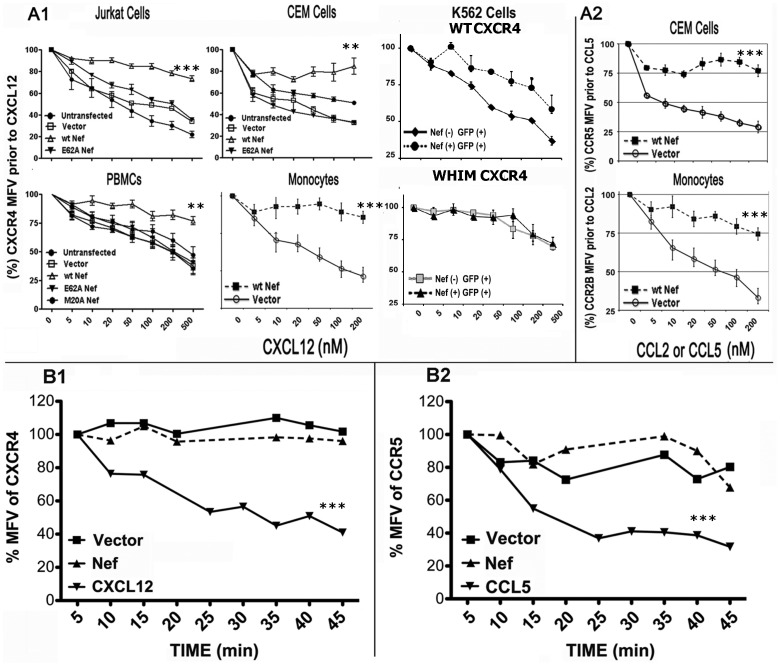
HIV Nef inhibits agonist mediated chemokine receptor internalization. Agonist dose response of wt or WM (WHIM syndrome) CXCR4 (**A1**), CCR2B or CCR5 (**A2**) clearance from the plasma membranes of Jurkat (CXCR4), CEM (CXCR4 or CCR5), K562 (wt or WM CXCR4) cells, fresh PBMCs (CXCR4), or monocytes (CCR2B or CXCR4) in the context of Nef expression. Cells were nucleofected (Amaxa Subdivision, Lonza Corp.) or not with a mixture of plasmids for GFP (for PBMCs) or CD8 (CEM, Jurkat and monocytes) and wt, some Nef mutants or null plasmids. In each case, ∼1×10^6^ transfected cells were treated in duplicate for 20 min with the indicated concentrations of CXCL12, CCL2 or CCL5. MFVs of CXCR4, CCR2B or CCR5 were determined by FACS analysis. Data are shown as relative mean fluorescent values (MFV) (%) of untreated sample(s) as a function of agonist concentration. The relative % downregulation was calculated after assigning receptor MFV in the absence of agonist to 100% for Nef (−) or Nef (+) cells. MFV data analysis was limited to GFP or CD8 gated cells. K562 cells were nucleofected with wt or WM CXCR4 and a bicistronic IRES plasmid encoding wt Nef or a null mutant and GFP. Data for Nef (−) & GFP (+) and Nef (+) & GFP (+) K562 cells expressing wt (top) or WM CXCR4 (bottom) were separately analyzed. For each transfection, data (in duplicate) for each cell population were used to fit a polynomial regression curve with standard deviation (n = 4). In A1 & A2, *** represents p<0.001, **p<0.01 when mean is compared with plasmid transfected cells. For experiments with Nef (−) K562 cells, ** represents p<0.01 when GFP positive cells were compared to GFP negative cells. Nef did not significantly enhance the intrinsic (non-agonist driven) internalization rates of CXCR4 (**B1**) or CCR5 (**B2**). CEM-NKR cell line expressing CCR5 and CXCR4 was nucleofected with GFP and Nef or null plasmid. At 16 h post-nucleofection, cells were stained (at 1×10^7^cells/ml) in RPMI with 2% FBS and containing unlabeled CCR5 (2D7) or CXCR4 (12G5) mAb at 4°C for 15 min. They were then shifted to 37°C, and left untreated or treated with 100 nM CCL5 or CXCL12 (vector and GFP co-transfectants only) at 37°C. At each indicated time point, aliquots were shifted to 4°C, washed thrice with 10× volumes of RPMI and the amount of bound antibody at the cell surface visualized and quantified in a flow cytometer after staining with Alexa 647 conjugated goat anti-mouse antibody (Invitrogen Corp). Each point (for GFP gated cells) is the mean of duplicate MFVs, expressed relative to MFV at time zero, which was arbitrarily set in each case to 100%. The MFV plots of GFP gated cells represent averaged results of three experiments. (*** indicates p<0.05 compared to vector transfected cells for all time points after 15 min). All data in this figure are presented as mean ± standard deviation.

We then inquired whether HIV Nef enhanced CKR endocytosis by bona fide agonists, by recruiting alternative ligands or by accelerated constitutive endocytosis. To obtain a quantitative measure of receptor clearance in the control versus Nef transfectants, we compared the agonist dose-response curves of internalization of CXCL12 bound CXCR4 in PBMCs, Jurkat and CEM cells expressing native CXCR4 or K562 transfectants co-expressing wt CXCR4 with GFP and wt or the indicated Nef mutants or empty vector. Nef expression reduced the steady-state levels of the respective receptors and derivatives by 50–60%. WT Nef, but not the alanine substitution mutants at M20 or 62EEEE65 inhibited agonist mediated clearance of residual receptor(s) in all cells (Figure 1A1). Likewise, Nef inhibited CCL2 or CCL5 mediated clearance of CCR2B or CCR5 in fresh monocytes or a CEM cell line co-expressing GFP and Nef or null plasmid (Figure 1A2). Agonist driven CCR5 endocytosis shown in Fig. 1 used a CEM cell line over-expressing CXCR4 and CCR5 [51]. Previously we showed that agonist driven endocytosis of CCR5 was sluggish in human primary lymphocytes and HEK293 or HOS cell lines expressing CCR5 [49]. However, CCR5 endocytosis rate was comparable to that of CXCR4 in the CEM cell line as was also shown for CHO cell lines [61], [62]. Nef did not affect the poor internalization of the naturally occurring WHIM CXCR4 mutant [63], [64], [65] after agonist binding (Figure 1A1).

Nef enhances the intrinsic endocytosis rates of many receptors, notably, CD4, MHC I and II and CD28 [7], [8], [9], [49], [50], [66]. Recent work has shown that HIV and SIV Nef induce a modest increase in the constitutive internalization rates of CCR3 and CCR5 in a CHO cell line [21], [22] and CXCR4 in HEK293 transfectants [20]. However, we found that Nef expression induced no significant increase in the intrinsic endocytosis rates of either CXCR4 or CCR5 in a CEM cell line (Figure 1B1 and B2) or CXCR4 in Jurkat cells (data not shown). While it's possible that bound antibodies may induce conformational changes in the cognate receptor [67], our previous work [46], [47], [48], [50], [68] identified the antibodies used here as not impinging on agonist binding or signaling. Thus, it is more likely the reduction in the steady-state levels of PM CXCR4 or CCR5 in Nef expressing cells might reflect receptor sequestration during transit to the PM or abortive receptor recycling.

### Nef mediated CXCR4 downregulation is dependent on ubiquitinylation

**Figure 2:**
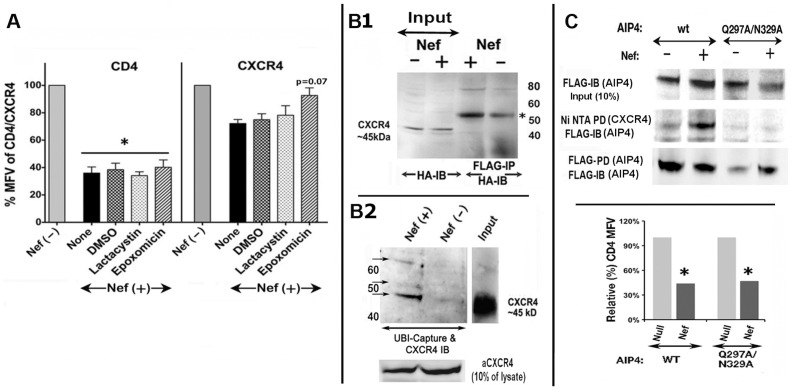
Nef induces CXCR4 ubiquitinylation mediated by the E3 ubiquitin ligase AIP4. A) Effect of proteosome inhibitors on Nef induced downregulation of CXCR4. Histograms showing relative (%) MFVs (with standard deviation) of native CXCR4 or CD4 (* p<0.01) in GFP gated Jurkat cells co-expressing GFP with Nef or null mutant and treated with the proteosome inhibitors lactacystin and epoxomicin at 25 µM (n = 4). **B**) Nef expression enhanced CXCR4 ubiquitinylation. HEK-293 cells were co-transfected with CD8, HA tagged CXCR4 and FLAG tagged ubiquitin with Nef or empty vector. Cytoplasmic extracts were prepared and an aliquot immuno-blotted to estimate HA-CXCR4 in the Nef (−) and Nef (+) transfectants (input lanes in B1). Ubiquitinylated proteins in the Nef (−) and Nef (+) cell lysates were affinity purified using FLAG mAb and immuno-blotted for CXCR4 using antisera against the HA epitope (**B1**). Molecular mass markers are denoted to the right of the gel in B1. Alternatively, ubiquitinylated proteins from Jurkat cells transfected Nef or null plasmid were captured using UBI-capture beads (Enzo Biochem) and resolved by SDS/PAGE followed by immuno-blot detection using anti-CXCR4 antibody (**B2**), Numbers to the left refer to molecular mass markers. The gel strip at the bottom illustrates immuno-blot analysis of aliquots (10%) of lysates (Input) for total CXCR4 content. (**C**) Nef recruits AIP4 to CXCR4 in the absence of receptor activation. Jurkat cells were co-tranfected with 6-His tagged CXCR4, GFP, FLAG-tagged wt or the Q297A/N329A AIP4 mutant that does not bind to CXCR4 [87], and Nef or a null plasmid. Cytoplasmic extracts were prepared after adjusting cell numbers to reflect equivalent GFP expression and aliquots were analyzed by SDS/PAGE and immuno-blot detection of AIP4 content using FLAG mAb (upper row). 6-His tagged CXCR4 proteins in the remaining extract were recovered by binding to Ni^++^ NTA, resolved by SDS/PAGE, and immuno-blotted with FLAG mAb for detecting AIP4 (middle row). To assess transfected levels of AIP4, cell lysates were immuno-precipitated with rabbit polyclonal antibody against AIP4 followed by immuno-blotting with FLAG mAb (lower row). Nef functionality was evaluated by monitoring CD4 expression on transfected cells by flow cytometry (histogram below, * p<0.01).

Similar to other GPCRs [43], [69], agonist-bound CXCR4 is ubiquitinylated by the E3 ligase AIP4 and targeted for lysosomal degradation [44], [70]. Initially, we examined the effect of proteosomal inhibitors on Nef mediated downregulation of CD4 or CXCR4 in Jurkat cells. Whereas neither lactacystin nor epoxomycin treatment restored CD4 levels in Nef transfectants, epoxomycin induced a modest reversal (from 68±5% to 93±7%, n = 4, p = 0.07, which is statistically not significant) of Nef effect on CXCR4 (Figure 2A). We next examined whether CXCR4 is ubiquitinylated in Nef expressing cells. HEK293 cells were cotransfected with HA CXCR4, FLAG-tagged ubiquitin and Nef or null vector. Cytoplasmic extracts were immuno-precipitated for Ubi-flag proteins and bound proteins were resolved by SDS/PAGE followed by immuno-blot detection of HA -tagged CXCR4. Nef expression led to increased (∼4 fold) detection of a prominent band of mono-ubiquitinylated CXCR4 (Figure 2B1). Alternatively, Jurkat cells were co-transfected with GFP and either Nef or empty vector. Cytoplasmic extracts were prepared and the ubiquitinylated internal CXCR4 were recovered using a commercial kit that captures specifically the ubiquitinylated proteins in the lysate (UbiTrap, Enzo Biochem). The bound proteins were resolved by SDS/PAGE followed by immuno-blot detection of CXCR4. In Figure 2B2, three protein bands of 50–72 kDa were observed in lanes corresponding to Nef expressing cells. Their molecular masses relative to unmodified CXCR4 of ∼45 kDa, (input) suggested that 1–3 (with one being prominent) ubiquitin molecules were conjugated to CXCR4. Such differences in the number of conjugated ubiquitins have been observed [70], [71] in different cell types. Furthermore, it was shown recently that a clear decision between mono- and poly-ubiquitinylation is made by the E3 ligase(s) in combination with specific accessory factor(s) [72].

Agonist binding induces direct interaction between CXCR4 and AIP4, presumably between phosphorylated serine residues in CXCR4 C-tail and the conserved WW domains of AIP4. Furthermore, Bhandari et al identified two conserved residues in the WW domain I (Q297) and II (N329) of AIP4 that are critical for binding CXCR4. CXCR4 behaves in an analogous manner in Nef expressing cells [63]. In the co-precipitation assay, Nef induced marked increase in the binding of AIP4 with CXCR4 and alanine substitutions at Q297 and N329 of AIP4 eliminated this binding (Figure 2C). CD4 down regulation in the transfected cells are shown to indicate the expression levels of Nef that down regulated CD4.

### Nef downregulated C-terminally truncated CXCR4s lacking putative GRK phosphorylation

**Figure 3:**
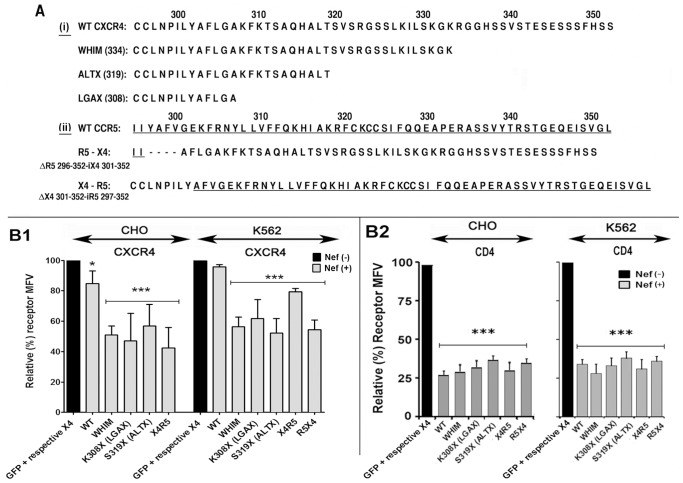
Cell surface expression of C-terminally truncated CXCR4 mutants and CXCR4/CCR5 chimeras were downregulated by Nef almost as well if not better than their wild type counterparts. A) C-terminal sequence coordinates of the chemokine receptors (i) C-terminal sequence of wt CXCR4 and selected C-terminally truncated mutants, including the natural WHIM mutant, numbers within parentheses denote the position of respective deletion; (ii) C-terminal sequence of wt CCR5 and CCR5/CXCR4 chimeras swapping the respective C-terminal sequence. The swap position(s) are denoted on the left. In each case, CCR5 sequence is underlined. **B**) Nef effect on genetically engineered CXCR4 mutants, the naturally occurring WHIM mutant (WM CXCR4) and chimeras (X4R5 and R5X4) was evaluated in CHO cells or CXCR4 negative K562 cell line. CD4 was cotransfected in each case to monitor Nef effect (**B2**). Cells were transfected with an IRES plasmid encoding GFP and Nef or the null mutant. Expression of CXCR4 or CCR5 (in the case R5X4 chimera) and CD4 in GFP gated cells was evaluated by flow cytometry. Average MFVs for CXCR4 (and CCR5 for R5X4 transfection) and CD4 on null and Nef (+) cells are plotted in the histograms (with standard deviation) for CXCR4 (and CCR5) expression in the left panel and for CD4 in the right panel. MFVs for Nef (−) cells were set to 100 (n = 4). *** represents p<0.01) when Nef transfected cells are compared to plasmid transfected controls. Black bars indicate relative % downregulation in Nef (wt or mutant) expressers relative to control cells transfected with empty vector.

Deletion or substitution mutants of CCR5 and CXCR4 devoid of motifs critical for constitutive and/or ligand-driven receptor endocytosis were still susceptible to Nef-mediated reduction of receptors at the cell surface [21]. We extended these observations by surveying Nef effect on C-terminally truncated CXCR4 including the naturally occurring WHIM CXCR4 mutant [63], [64], [65] and receptor X4/R5 chimeras swapping the respective C-tails of CXCR4 and CCR5 (X4-R5 and R5-X4) [46]. Since CXCR4 and derivatives were untagged, we evaluated their response to Nef in K562, which have substantially reduced levels of native CXCR4 and in CHO cells lacking human CXCR4. As illustrated by Figure 3, C-terminal truncations including the natural WHIM CXCR4 or genetically engineered deletions such as ALTX at 319 or LGAX at 308 and the CXCR4/CCR5 chimera were downregulated by Nef in both cell types (Figure 3).

### Lys residues in the C-tail of CXCR4 and the catalytic Cys residue in the HECT domain of AIP4 are critically required for Nef induced downregulation of CXCR4

**Figure 4:**
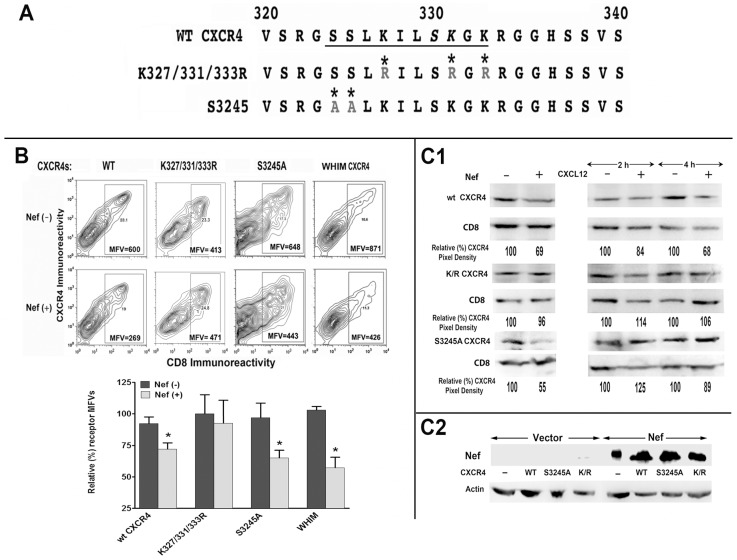
CXCR4 mutated at three lysines to arginines (K327/331/333R) was neither internalized nor degraded in Nef expressing cells, unlike the S3245A CXCR4 mutant that was readily degraded in Nef expressing cells. A) C-terminal sequence coordinates of wt, K327/331/333R and S3245A CXCR4 mutants are shown with the critical LYS/ARG and SER/ALA mutations denoted by asterisks The underlined residues indicates the degradation motif of CXCR4. **B**) Bivariate FACS analysis of HEK-293 cells transfected with CD8, HA-tagged wt or selected CXCR4 mutants with Nef or null vector. CXCR4 MFVs are shown within the respective quadrants (B, top). Average MFVs from four experiments are presented as histograms (with standard deviation) on the right (n = 4; * p<0.05), after adjusting the population to equivalent numbers of CD8 expressers. (B, bottom). **C**) Extracts of wt Nef (+) or null (−) transfectants or from cells treated with or without 100 nM CXCL12 for 2 or 4 h were immuno-blotted for detecting HA-tagged CXCR4 and CD8. Pixel densities of the respective CXCR4 bands were determined by scanning and normalizing to constant CD8 expression. Average pixel values from three experiments are shown relative to the respective controls. (**C2**) Nef expression in the respective wt and mutant CXCR4 transfectants was evaluated by immuno-blotting and normalizing to actin levels.

A degradation motif (324SSLKILSKGK333) near the C-terminus of CXCR4 containing three lysines is targeted for ubiquitinylation that is required for receptor degradation. Serines at 324, 325, and possibly 330, whose phosphorylation by GRKs following agonist binding facilitates ubiquitinylation of the aforesaid lysines [44]. We observed that arginine substitutions at the three lysines (K327/331/333) rendered CXCR4 insensitive to Nef induced clearance from the plasma membrane (Figure 4B). However, mutant(s) substituting the two serines (S324 & S325) with alanines were still downmodulated by Nef. Interestingly, the naturally occurring WHIM mutant with a C-terminal truncation, which spared the degradation motif was also down modulated by Nef (Figure 4A & B). Steady state levels of these mutants after agonist treatment or during Nef expression were evaluated by immuno-blotting. While the triple Lys/Arg mutant was not degraded by agonist treatment, or Nef expression, the S324A/S325A mutant was degraded by Nef, but not after agonist (CXCL12) treatment (Figure 4C1).

**Figure 5:**
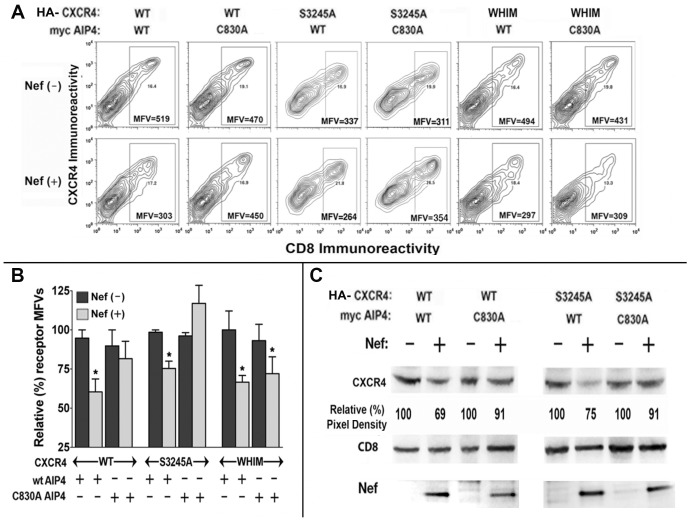
Nef induced downregulation of CXCR4 was critically dependent on the functional integrity of the HECT domain of AIP4 sub-served by the cysteine residue at position 830. A) Bivariate (CD8 versus CXCR4) expression profiles of Nef (−) and Nef (+) HEK-293 cells co-transfected with CD8, HA-tagged wt, S3245A or WHIM CXCR4 and Nef or null vector in the context co-expression with c-myc tagged wt AIP4 or C830A AIP4 mutant lacking E3 ligase activity are illustrated by the set of contour diagrams. **B**) CXCR4 MFVs shown within the gated regions of the respective contour profiles from four experiments were averaged, normalized for equivalent CD8 expression and plotted as histograms (with standard deviation; n = 4; *, p<0.05). **C**) Steady-state levels of HA-tagged wt or S3245A CXCR4 in Nef (−) versus Nef (+) cells co-expressing wt or C830A AIP4 were detected by immuno-blotting. Pixel densities of CXCR4 bands were determined by scanning and normalizing to constant CD8 expression and average pixel values from three experiments is shown. CD8 and Nef immuno-blots for the corresponding transfections are illustrated underneath. WT and C830A AIP4 were detected by c-myc immuno-blotting, but not shown.

AIP4-C830A, which is a catalytically inactive mutant of AIP4 markedly reverses agonist-promoted degradation of CXCR4 [44]. AIP4-C830A also reversed the Nef induced down modulation of WT and S324A/S325A CXCR4 mutant and the degradation of the WT receptor (Figure 5A & 5B). However, AIP4-C830A mutant did not rectify Nef induced downregulation of WHIM CXCR4 (Figure 5A & 5B), which has three critical lysines, and presumptive serine targets of GRK phosphorylation at 324, 325 and 330.

### Nef sequesters CXCR4 in perinuclear vesicles and co-localizes with AIP4, NEDD4, E3 ligases, ESCRT-0 adapter(s) and endolysosomal markers

**Figure 6:**
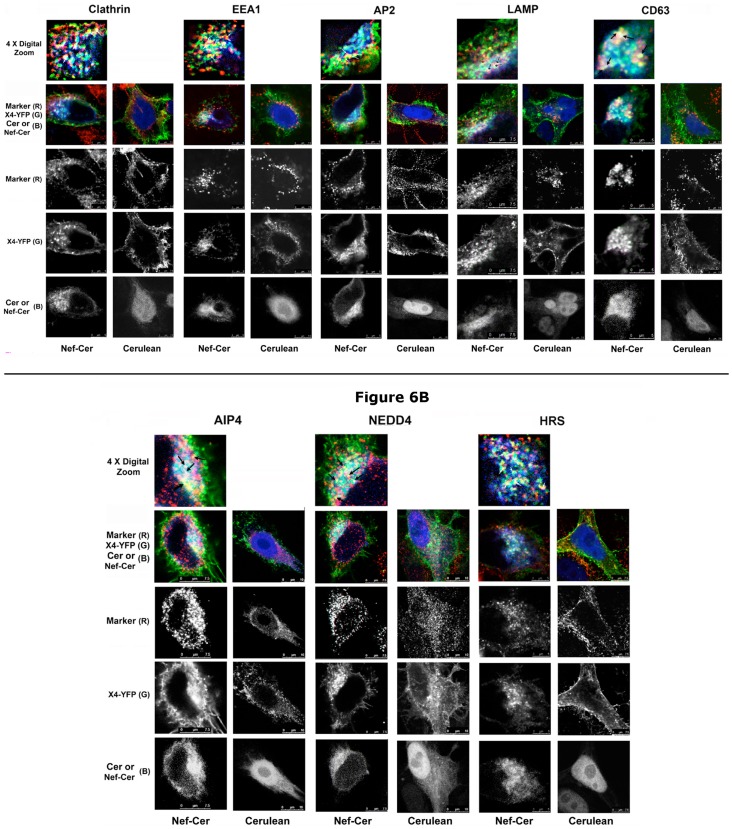
Subcellular distribution of CXCR4-YFP in HeLa transfectants co-expressing Nef-Cer or Cer. Host endo-lysosomal markers, E3 ubiquitin ligases AIP4 and NEDD4 and HRS were identified by primary antibody staining (as described under Methods) followed by Alex-647 conjugated secondary antibodies [47]. Confocal images corresponding to Nef-Cer and Cer transfectants co-expressing CXCR4-YFP are shown pairwise with co-staining for clathrin, EEA1, AP2, LAMP and CD63 in Figure **6A**. Similar results for costaining AIP4, NEDD4 and HRS are shown in Figure **6B**. Individual channels corresponding to the respective cellular proteins (R), CXCR4-YFP (G) and Nef-CerFP or Cer (B) fluorescence are shown below the composite RGB images. 4-X cropped images of Nef-Cer transfectants are shown in the top row of each figure, with the arrows denoting colocalization of the respective indicated proteins. 7.5 or 10 µm scale bars are shown. Results are representative of three independent experiments.

Steady-state distribution of YFP tagged CXCR4 was examined in cells co-expressing Nef-CerFP or Cer. In the Nef-CerFP (+) cells, substantial CXCR4-YFP colocalized with Nef-CerFP in the perinuclear vesicles with the remaining receptor distributed in a speckled pattern near the plasma membrane (Figure 6A and Figure S3). In the absence of Nef, CXCR4-YFP was predominantly, if not exclusively distributed at the plasma membrane (Figure 6A and Figure S3). Transfectants were also stained with antibodies against the following subcellular organelles: clathrin, early endosomal marker EEA1, late endosomal and lysosomal markers, CD63 and LAMP, ESCRT-0 adapter Hrs/Vps27 (hepatocyte growth factor-regulated tyrosine kinase substrate) and E3 ubiquitin ligases AIP4 or NEDD4. It has been shown before that following agonist treatment, internalized CXCR4 colocalized with late endosomes or lysosomes, rather than early endosomal marker(s) [38]. Likewise, we observed in Nef expressing cells, CXCR4 was sequestered in very few clathrin-coated vesicles (CCVs) or AP2 and EEA1 enriched vesicles. More extensive co-localization of CXCR4 and Nef was noted with the CD63 and LAMP-1 positive vesicles (note the marked increase in the number of yellow, orange and white vesicles in Figure 6A, top row). There was also marked co-localization with the HECT domain E3 ligases (AIP4 and NEDD4) and Hrs/Vps27 positive ESCRT-0 structures (Figure 6B).

### Differential effects of siRNA knockdowns of vesicular and ESCRT adapters and E3 ligases on Nef induced loss of chemokine receptors

**Figure 7:**
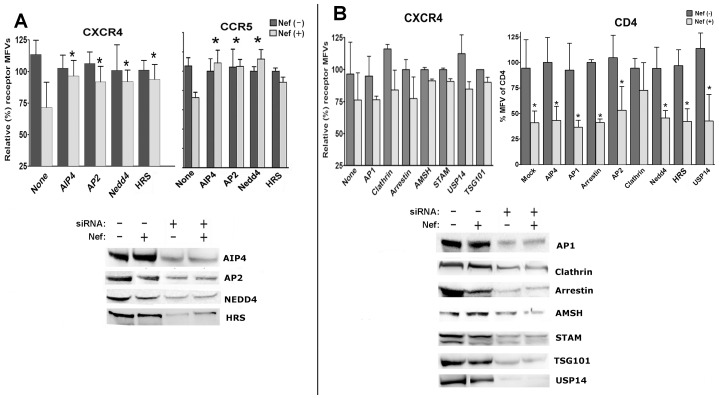
siRNA knockdown of AP2, E3 ubiquitin ligases, AIP4 and NEDD4 and Hrs/Vps27, a candidate ESCRT-0 protein reversed Nef induced downregulation of CXCR4 or CCR5. **A**) Histograms of relative (%) MFVs (with standard deviation) of native CXCR4 (left) in Jurkat cells or CCR5 in CEM cell line (right) expressing Nef and GFP are shown in the context of siRNA knockdown of AIP4, AP2, NEDD4 and HRS (*p<0.05 compared with Nef and mock siRNA transfected cells). **B**) Nef induced CXCR4 downregulation was not reversed by siRNA knockdown of AP1, clathrin, β-arrestin, deubiquitinases, AMSH, STAM, and USP14 and a candidate ESCRT adapter, TSG101/Vps23P. CD4 downregulation by Nef was resistant to all siRNA knockdowns except for clathrin (**B**, right, n = 5; *p<0.01). Expression levels of proteins targeted by cognate siRNAs were monitored by immuno-blots shown underneath panels **A** and **B**.

We compared the effect of siRNA knockdown of various endocytosis adapter molecules on HIV-1 Nef induced downregulation of endogenous CXCR4 and CCR5 in Jurkat and CEM cells subunits, siRNA knockdown of clathrin, βarrestin-2, ESCRT-0 components STAM (signal transducing adapter molecule), Hrs/Vps27 and TSG101/Vps23P (tumor susceptibility gene 101), HECT domain E3 ligases and AMSH (Associated Molecule with the SH3-domain of STAM, which functions as a deubiquitinase *in vitro*) or USP14 (Ubiquitin carboxyl-terminal hydrolase- 14, which is a member of the ubiquitin-specific processing (UBP) family of proteases that is a deubiquitinating enzyme) on Nef induced downregulation of endogenous CXCR4 and CCR5 in Jurkat and CEM cells. The various siRNAs induced a marked loss of the cognate proteins (Figure 7). Among all the siRNA knockdowns, only a reduction of AIP4 or NEDD4 E3 ubiquitin ligase, ESCRT-0 component Hrs/Vps27, or the AP2 adapter was effective in restoring the plasma membrane levels of CXCR4 and CCR5 in Nef expressing cells. For instance, knockdown of AIP4 reversed the Nef effect on CXCR4 in Jurkat cells from 74±5% to 96±6% and on CCR5 in CEM cells from 76±6% to 106±8% of control. AP2 α chain knockdown induced an increase in CXCR4 MFV (mean fluorescence value) from 74±5% to 90±5% and CCR5 from 74±5% to 102±6%. While ablation of NEDD4 E3 ligase reversed Nef effect on CXCR4 or CCR5 levels by similar magnitudes, knock down of Hrs/Vps27 partially rectified Nef effect on CXCR4 but not CCR5 (Figure 7A). Knockdown of other ESCRT and endocytosis adapters induced marginal and/or statistically inconsistent results (Figure 7B). siRNA knockdowns against other targets did not have any effect on Nef mediated CD4 down regulation except for clathrin, which was demonstrated to participate in CD4 downregulation (Figure 7B). Although other reports have shown that Nef effect on CD4 was partially remedied by AP2 knockdown [73], [74], [75], we have shown here and elsewhere [49], [50] that AP2 knockdown was more effective in reversing the effect of SIV than HIV Nef on CD4. Apropos SIV Nef, we wish to reiterate sour statement in an earlier paper [49], [50] SIV Nef being endowed with both a canonical Y*XX*Ø sequence near the N terminus and a EEHY**LM**HPA sequence (where the -LM- dipeptide is highlighted in bold and the dileucine motif equivalent is underlined) near the C terminus, binds to the AP-2 adaptor more vigorously that HIV Nef. That being said, it is possible that AP2 knockdown was not complete in our case, which might account for the discrepancy with other results.

### Nef induced downregulation of CXCR2 was not dependent on ubiquitinylation

**Figure 8:**
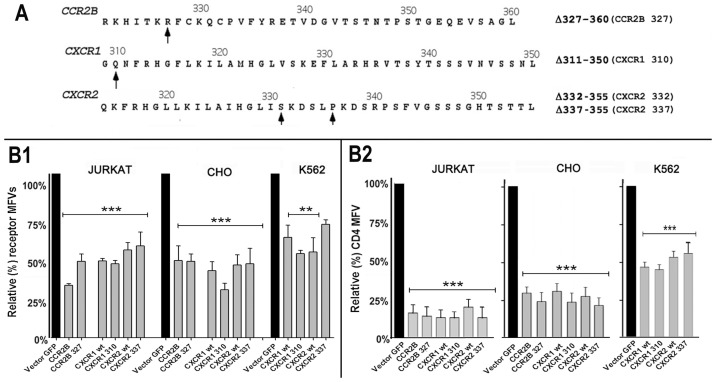
Nef downregulated cell surface expression of wt and C-terminally truncated CCR2B, CXCR1 and CXCR2 receptors in T cell line and epithelial cells. A) C-terminal sequence coordinates of CCR2B, CXCR1 and CXCR2 are shown with the arrows denoting the C-termini of the respective truncation mutants. **B1**) Nef effect on wt and C-terminally truncated derivatives of CCR2B, CXCR1 and CXCR2 was evaluated in Jurkat, CHO and K562 cells. Cells were co-transfected (nucleofection of Jurkat and K562 cells and lipofection of CHO cells) with expression plasmids for the indicated receptors and IRES plasmids encoding Nef and GFP or a null Nef mutant and GFP (vector). For all the comparisons of receptor levels between the Nef and plasmid transfected cells, the p value was less than 0.05 (**). **B2**) CD4 expression plasmid was introduced in CHO and K562 transfectants to monitor Nef effect (***p<0.01). Cell surface expression of receptors was analyzed and data presented as in Figure **3 B** (n = 4).

We compared the Nef effect on the steady-state levels of wt and C-terminally truncated CCR2B, CXCR1 and CXCR2 receptors to inquire whether ubiquitinylation dependent endocytosis was a consensual mechanism. As illustrated by Figure 8 B1, Nef expression downregulated C-terminally truncated CXCR1, CXCR2 and CCR2B mutants almost to the same extent as their wt counterparts in lymphoid and epithelial cells.

**Figure 9:**
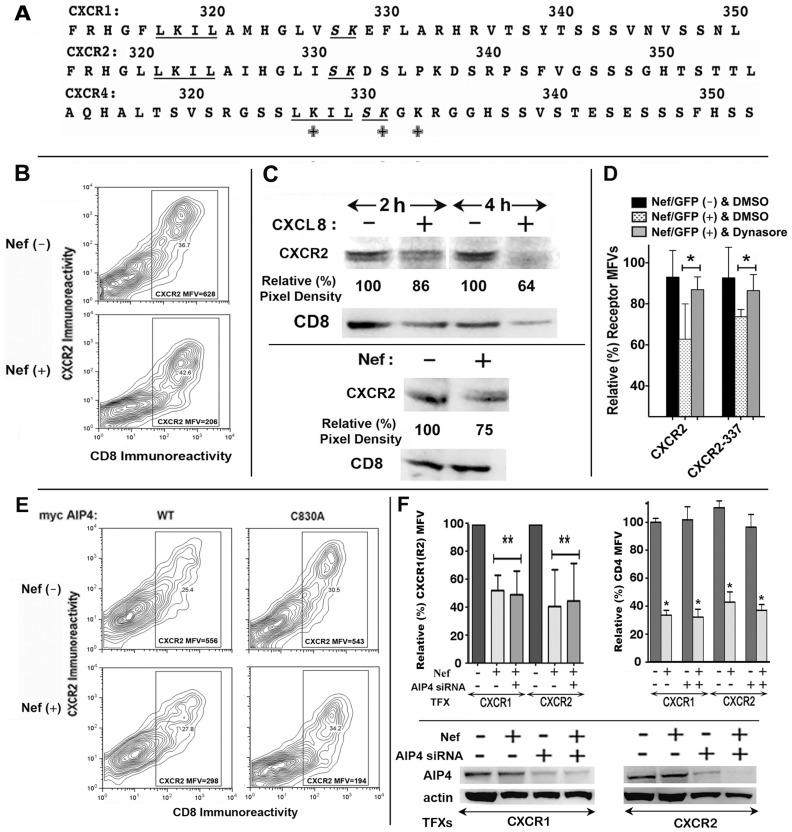
Nef induced downregulation of CXCR2 was not dependent on ubiquitinylation. A) Amino-acid sequence of C-termini of CXCR1, CXCR2 and CXCR4 are shown, with the lysines critical for CXCR4 ubiquitinylation denoted by **+**, and the lysine motifs conserved in CXCR1, CXCR2 and CXCR4 by underlined italicized text. **B**) Bivariate (CD8 versus CXCR2) FACS contour analysis of HEK-293 cells transfected with expression plasmids for CD8, CXCR2 and Nef or a null vector. CXCR2 MFVs are shown within the respective quadrants. Results are representative of three HEK-293 transfections. **C**) Nef expression or agonist treatment led to physical loss of CXCR2. HEK-293 cells were cotransfected with CD8 and CXCR2 and a Nef or a non-Nef plasmid. Cells were treated with 100 nM CXCL8 for 2 or 4 h. Total CXCR2 was detected by immuno-blotting. Average pixel values from three experiments are shown relative (%) to the respective untreated controls. CXCR2 in Nef (−) versus Nef (+) cells are shown below the results of CXCL8 treatment. Representative CXCR2 immuno-blots of transfectants are shown pair wise for Nef (−) & Nef (+) cells. Cell numbers in each pair were normalized to constant CD8 expression detected by immuno-blotting as illustrated. Relative CXCR2 (%) pixel values are averages from three transfections. **D**) Dynasore treatment reversed Nef mediated downregulation of wt CXCR2 and to a less extent CXCR2 337 (sequence coordinates in Fig. 8). Jurkat cells were cotransfected with GFP, Nef and wt or CXCR2 337. Cells were treated with 80 µM dynasore in DMSO or equivalent DMSO alone for 4 h prior to analysis of CXCR2 expression by flow cytometry. Histograms (with standard deviation) represent relative (%) receptor MFVs in Nef (+) versus Nef (−) cells (n = 3, *p<0.05). **E**) Unlike CXCR4, Nef induced downregulation of CXCR2 was not reversed by over-expressing E3 ligase negative C830A AIP4 mutant. HEK293 cells were co-transfected with a mixture of CD8 and CXCR2, myc-tagged wt or C830A AIP4 and Nef or a null plasmid. Bivariate FACS contour plot of cell surface CD8 and CXCR2 showing that Nef induced loss of cell surface CXCR2 irrespective whether wt AIP4 or functionally negative C830A mutant was co-expressed. CD8 transfection efficiency and CXCR2 MFVs are shown within the relevant gates. **F**) siRNA knockdown of E3 ubiquitin ligase, AIP4 failed to reverse Nef induced downregulation. Jurkat cells were nucleofected with the indicated siRNAs 36 h prior to DNA transfection with CXCR1 or CXCR2 and plasmids for GFP and Nef or null plasmid. Cell surface expression of CXCR1 and CXCR2 in GFP gated Nef (−) and Nef (+) cells were quantified by flow cytometry. Histogram on the left shows relative (%) MFV (with standard deviation) for CXCR1 or CXCR2 in Nef/GFP (−) versus Nef/GFP (+) cells in the context of siRNA knockdown of AIP4. AIP4 ablation had no effect on Nef induced CD4 downregulation as illustrated by the histogram on the right (n = 3; p<0.05). AIP4 knockdown was verified by immunblotting the extracts from the respective transfections as illustrated underneath the histograms.

Among the CXC receptors, CXCR1 and CXCR2 display homology with CXCR4 in their carboxy-terminal domains as reflected by the conserved –LKIL- and SK- motifs containing lysine residues, which are critical for ubiquitinylation of agonist-occupied CXCR4 (Figure 9A). Nef expression induced a significant loss of CXCR2 from the cell surface (Figure 9B & 9C) and marked degradation of steady state levels of CXCR2 (Figure 9C, bottom) as observed after prolonged agonist (CXCL8) treatment (Figure 9C, top). We have shown before that internalization of agonist occupied CXCR1 and CXCR2 was sensitive to inhibition of dynamin and clathrin adapters such as Eps15 [70]. As expected, dynasore substantially reversed the Nef induced clearance of wt CXCR2 and to a less extent C-terminally truncated CXCR2-337 mutant from the cell surface (Figure 9D).

Lysosomal degradation of CXCR2 following protracted agonist treatment was shown not to require receptor ubiquitinyation [71], but modulated by the C-terminal type I PDZ ligand motif of CXCR2 [76]. We inquired whether Nef induced CXCR2 degradation was ubiquitinylation dependent, requiring recruitment of AIP4. Nef effect was evaluated in HEK-293 cells expressing CD8 and CXCR2 with or without the catalytically inactive AIP4-C830A. Unlike what was observed with CXCR4, AIP4-C830A expression did not alter Nef induced CXCR2 downregulation (Figure 9E). We also compared the effect of siRNA knockdown of HECT domain E3 ligases on Nef induced loss of CXCR1 and CXCR2. Knockdown of AIP4 failed to counteract the Nef effect on CXCR1 or CXCR2 in Jurkat cells. After AIP4 knockdown, relative MFVs in Nef expressing cells went from 53±3% to 47±4% for CXCR1 and from 39±5% to 41±6% of control for CXCR2 (Figure 9 F). Similar results were obtained with NEDD4 knockdown (not shown). While it is possible that, Nef recruits other unidentified E3 ligases, our results suggested that Nef mediated downregulation of CXCR1 and CXCR2 may share the same script as that of agonist (CXCL8 in this case) treatment described before.

## Discussion

HIV Nef protein is a versatile modulator of intracellular trafficking of many immune cell receptors including multiple chemokine receptors. This versatility reflects the redundant mechanisms that Nef uses to recruit multiple vesicular transport adapters. Nef is presumed to act as a connector between receptors and endocytic machinery through components of CCVs and vesicular adapter complexes [14], [50], [55], [77], [78], [79], [80]. Through multiple criteria we have shown that for CXCR4, and probably CCR5, Nef exploits a slightly different strategy. Nef usurped a well established and physiologically relevant mechanism of lysosomal sorting of agonist occupied GPCRs through ubiquitinylation [43], [44], [69], [70]. Nef induced CXCR4 ubiquitinylation under basal condition by direct recruitment of AIP4, a HECT domain E3 ligase without requiring agonist stimulation or receptor phosphorylation.

Although it has been known that Nef proteins of different primate lentiviruses downregulate multiple CKRs, with SIV Nef in general being the most potent [20], [21], there is limited understanding of the mechanism(s) of downregulation. It has been suggested that Nef accelerates CXCR4 endocytosis via an AP2 dependent pathway since Nef mutants lacking AP2 adapter binding potential lost the phenotype [20]. Analysis of the effects of Nef on a large number of CKRs and mutant derivatives led to the conclusion that Nef modestly accelerated basal or agonist-driven endocytosis without the need for classical endocytosis motifs in the CKR cytoplasmic tail, for heterotrimeric G protein binding, or Gα_i_ signaling [21]. We found that Nef downregulated CKRs to a variable degree in many cell types. Our findings indicated that Nef does not increase the intrinsic endocytosis of unoccupied CKRs, but rather establishes a new set point for receptor expression by perturbing receptor re-cycling thereby favoring receptor degradation. The residual plasma membrane CKRs in Nef (+) cells were recalcitrant to agonist induced internalization.

Since the original discovery of K63-linked mono-ubiquitinylation as the receptor endocytic signal in yeast [81], [82], [83], many plasma membrane receptors, including both single transmembrane growth factor receptors and GPCRs have been shown to be ubiquitinylated on one or more lysines prior to ESCRT coupled traffic to lysosomes for degradation [84], [85], [86]. CXCR4 has been a prototype CKR in the quest to understand the mechanism of ubiquitin regulated lysosomal sorting of a mammalian GPCRs. Agonist stimulation of CXCR4 leads to ubiquitinylation of one or more Lys residues within the C-terminal ^324^SSLKILSKGK^333^ sequence by HECT domain E3 ubiquitin ligase, AIP4 through physical interaction with the receptor [70], [87]. Likewise, we found that AIP4 was recruited to CXCR4 in Nef expressing cells resulting in the addition of 1–3 ubiquitin molecules to the receptor although Nef itself was not ubiquitinylated (not shown) under these conditions. This was further substantiated by the partial reversal of Nef mediated CXCR4 downregulation by co-expression of catalytically inactive AIP4-C830A mutant.

Previous work has shown that AIP4 ubiquitinylated CXCR4 at the plasma membrane in an agonist-dependent manner and upon internalization, CXCR4-AIP4 complex mediated CXCR4-dependent ubiquitinylation of Hrs [70]. By siRNA knockdowns, both AIP4 and Hrs were shown to be required for targeting CXCR4 to the lysosomal degradation [42]. siRNA knockdowns of AIP4 and Hrs/Vps27 led to a reversal of Nef effect suggesting that Nef mediated CXCR4 down-modulation followed essentially the same pathway. Besides AIP4, β arrestin-2 [88], CISK kinase [89], members of ESCRT pathway, such as AMSH [90], STAM-1 [91] and the deubiquitinase USP-14 [92] were shown to be involved in the agonist dependent sorting of CXCR4 to lysosomes. Whether these ESCRT pathway components play similar role(s) in the Nef induced CXCR4 itinerary was not clear from our siRNA knockdown experiments.

Nef induced binding of AIP4 to CXCR4 probably occurs as a ternary complex of CXCR4, Nef and AIP4. There was substantial co-localization of CXCR4 with Nef in AIP4 and NEDD4 enriched vesicles. CXCR4 and Nef preferentially co-localized in vesicles with late endolysosomal markers like CD63 and LAMP, rather than EEA1 and AP2 vesicles. Our confocal imaging being a snap shot during transient expression might accentuate colocalization at the final destination (i.e. lysosomes) of endocytic traffic rather than the intermediary transit points. However, we have shown substantial colocalization of Nef and CXCR4 with the HECT domain E3 ligases and the ESCRT 0 adapters that are the rate-limiting steps in the ubiquitin dependent ESCRT trafficking. Certain GPCRs, such as PAR retain both Ubq independent constitutive endocytosis and Ubq dependent agonist driven internalization [86]. Selection of a particular endocytic intinerary may be positively regulated by ubiqutin [86] or negatively regulated by the failure of association of Epsin and or other CLASPs containing ubiquitin interacting motifs (UIMs) with the CCVs [93], [94]. The partial colocalization of Nef and CXCR4 with the components of ubiquitin dependent and independent pathways reflects this inherent dichotomy. In this context, we wish to point out that wt CXCR4 downmodulation by Nef varied from 40% in PBLs to no downmodulation in K562 cells (Table 1) and this difference did not correlate with differences in AIP4 or NEDD4 expression (unpublished data).

Nef bound AIP4 in a specific and quantifiable manner, both *in vitro* in GST-Nef pull-down and *in vivo* in co-precipitation assays [95]. AIP4 has four WW domains, which preferentially bind to proline-rich PY motifs [96], [97], [98], hyper-phosphorylated C-termini, or acidic domains of plasma membrane receptors [87]. Accordingly, AIP4 mutated at the two conserved residues, Q297 and N329 of WW domains I and II failed to bind CXCR4 in Nef expressing cells as was shown following agonist stimulation [87]. Nef expression substantially enhanced *in vivo* binding of CXCR4 to recombinant AIP4 and this binding was lost for the Q297A/N329A AIP4WW domain mutant. Nef mutants with alanine substitutions at the tetra-glutamate (^62^EEEE^65^) domain or at the polyproline (^72^PXXP^75^) motif failed to downregulate CXCR4 and were also deficient in binding AIP4 *in vitro* or *in vivo* [95].

There were some remarkable differences between agonist and Nef mediated CXCR4 downmodulation. First, while AIP4 was the exclusive E3 ligase recruited to agonist-bound CXCR4, Nef recruited AIP4 and possibly NEDD4 (as suggested by co-localization experiments) to CXCR4, and siRNA knockdown of NEDD4 partially reversed the Nef effect. Second, unlike the degradation of CXCR4 following agonist treatment, which requires phosphorylation of CXCR4 serine residues 324 and 325 for AIP4 recruitment [44], [87], Nef readily degraded a receptor mutant, S324A/S325A that lacked those serines. AIP4 binding occurs between its WW domains and PPPY or PXXP motifs [97], [98] or phosphoserine and phosphothreonine(s) on its targets [99]. By directly recruiting AIP4 through its PXXP motif(s) [95], Nef serves as a “bridge”, bypassing the requirement of CXCR4 phosphorylation for AIP4 binding.

Nef down-modulated the naturally truncated WHIM CXCR4 mutant, which retains the ^324^SSLKILSKGK^333^ sequence with three critical lysines and two serines, but is nevertheless impaired for CXCL12 mediated endocytosis [63], [64], [65]. Nef effect on WHIM CXCR4 was not reversed either by over-expression of the catalytically inactive AIP4-C830A mutant or by siRNA knockdown of AIP4 (not shown). The K327/331/333 and the CXCR4 K308X (shorter than the previously described Δ316 mutant [21]) lack the critical lysines and therefore cannot be ubiquitinylated, while the WHIM mutant was not ubiquitinylated in Nef expressing cells. Thus, Nef induced ubiquitinylation requires critical lysines within a bona-fide C-terminal domain. Although we haven't evaluated binding parameters for CXCR4 truncations versus AIP4, Bhandari *et al* [87] have demonstrated that C terminal serines at 324/5 of CXCR4 to be important for AIP4 binding. Since ALTX and LGAX mutants lack this critical residue, we speculate that these mutants might not be able to bind to AIP4. Therefore, it would seem that a different E3 ligase may be recruited by Nef in the case of WHIM CXCR4, or more likely WHIM CXCR4 and other truncated derivatives lacking the critical lysines are directly routed to endosomes, possibly through association with adaptin 2 subunits via the –LKIL-motif. There is precedence for the latter proposal. A C-terminally truncated CXCR2 (at position 331) was not phosphorylated upon agonist binding, but was internalized. The CXCR2 mutant had reduced association with β-arrestin 1 but continued to exhibit association with adaptin 2 α and β subunits, through the LLKIL motif [100]. In the case of WHIM CXCR4, Nef might be enhancing an inherent weak interaction between the –LKIL- motif and AP2 subunit(s). However, we were unable to reverse Nef effect on CXCR4 by siRNA knockdown of AP2 (data not shown). Besides, dynasore treatment did not reverse the Nef mediated downregulation as it did for wt CXCR4, which suggested that the classical endocytosis pathway may not be operational for the WHIM mutant.

Although Nef downregulated numerous CKRs, we could only identify direct evidence for ubiquitinylation of CXCR4 and a circumstantial one for CCR5. Among the CXC receptors CXCR1 and CXCR2 retain the –LKIL- and –SK- motifs that are ubiquitinylation targets in CXCR4. However agonist-driven CXCR2 degradation does not require ubiquitinylation, but is mediated by the C-terminal PDZ ligand motif [76]. Likewise, we found that Nef induced CXCR1 and CXCR2 degradation did not require ubiquitinylation. Other GPCRs like DOR [101], PAR-1 [86], [102], or CXCR3 [103] are also sorted to lysosomes by mechanisms not requiring ubiquitinylation. Nef induced downregulation of CXCR4 truncation mutants, ALTX and LGAX, which lack both the –LKIL- and –SK- motifs, reflects a similar scenario. Thus, there is substantial variability and plasticity in the physiological sorting pathways of GPCR by endocytosis and it is possible that Nef exploits different pre-existing itineraries by recruiting critically relevant adapters.

In addition to the reduction in steady state levels of CKRs the Nef expressing cells also exhibited a significant defect in agonist induced receptor downregulation. Of the CKRs examined, the highest concentration of agonist triggered approximately 75% reduction in cognate receptor expression while the Nef expressing cells exposed to ligand retained approximately 80% of their initial cognate receptor expression. In some sense the Nef expressing cells transfected with wt CXCR4 resembled the control cells expressing the WHIM CXCR4 mutant. The WHIM CXCR4 expressing cells retained approximately 75% of their initial receptor expression following agonist exposure. Yet the outcomes are very different; WHIM CXCR4 receptor expressing cells exhibit hypersensitivity to agonist with enhanced intracellular calcium flux, while the Nef expressing cells are profoundly hypo-functional exhibiting very poor signaling following CXCL12 exposure. The slightly less than two fold reduction in the baseline CXCR4 expression in the Nef expressing cells cannot account for this marked reduction in signaling; rather Nef impaired heterotrimeric G-protein signaling by targeting Gα_i2_ for degradation [95] thereby resulting in calcium flux arrest and chemotaxis defects. Thus, Nef expression has not only impaired receptor recycling, but has also interfered with the ability of the remaining CXCR4 to engage downstream effectors.

## Supporting Information

Figure S1 **Recombinant Nef protein was taken up by lymphocytes and induced efficient downregulation of CD4 and CXCR4 in T lymphocytes.** A) Schematic diagram (top) of recombinant Nef protein with the Tat RRM (arginine rich motif) domain appended at the C-terminus followed by 6 His residues, which was co-expressed in *E. coli* with the yeast N-myristoyl transferase. N-myristoyl or unmyristoylated Nef-Tat RRM protein(s) in E. coli extracts were purified by metal-affinity chromatography by successive batch elution with 25 and 100 nM imidazole. Proteins were resolved by SDS/PAGE, blotted and detected by chemiluminescence using Ni^++^-HRP (bottom left). Nef-Tat RRM induced marked downregulation of CD4 and CXCR4 in Jurkat cells. Jurkat cells (5×10^2^/ml) were incubated for the indicated times with affinity-purified Nef-Tat RRM (5 µg) in 0.2 ml of serum free RPMI prior immunological detection of CD4 and CXCR4 by flow cytometry. Bivariate FACS profiles of CXCR4 and CD4 are shown on the right. Results are representative on four experiments. **B**) Nef reduced the plasma membrane density of CCR5 and CXCR4 in a CEM cell line in the context of HIV infection CEM cells were infected with 400 ng p24 equivalent VSV-G pseudotyped NL4-3 HIV per 10^7^ cells. HIVs used in this study express CD24 in place of VpR in a Nef (+) (CD24 wt) or a Nef (−) (CD24 M1T) background and have been described before [47]. Cells were harvested 24–36 h post infection. CD4, CCR5 or CXCR4 were detected using respective mAbs conjugated with APC.CD24 was detected using PE conjugated CD24 mAb. Bivariate FACS profiles of CCR5 or CXCR4 versus CD24 for uninfected, CD24 wt or CD24 M1T CEM cells with the respective receptor MFVs for the different gated populations are shown on the left. Results are representative of three experiments.
(TIF)

Figure S2 **Nef induced CXCR4 downregulation by Nef was critically dependent on the tetra-glutamate and the poly-proline motifs of Nef.** Effect of wt and mutant Nefs on native CXCR4 and CD4 was evaluated in Jurkat, CEM cells or fresh PBMCs (**A** and **B**). Cells were cotransfected with the indicated expression plasmids and a reference CD8 (Jurkat and CEM) or GFP (PBMCs) plasmid for gating. Histogram bars represent arithmetic means of MFVs, plotted with standard deviation (@n = 4, p<0.02; *n = 3, p<0.04).
(TIF)

Figure S3 **CXCR4 co-localizes with Nef in the perinuclear region: confocal microscopy was done on Hela cells transfected with CXCR4 YFP and Nef CerFP or Cer.** Individual channels corresponding to CXCR4-YFP (G) and Nef-CerFP or Cer (B) fluorescence are shown alongside to the composite RGB images. Five representative fields with 7.5 or 10 µm scale bars are shown.
(TIF)

Procedures S1 **Bacterial expression of GST-Nef fusion protein.**
(DOCX)
