# Supplementary figures and images for: HIV-1 Nef Down-Modulates C-C and C-X-C Chemokine Receptors via Ubiquitin and Ubiquitin-Independent Mechanism
Source: PLoS One. 2014 Jan 29;9(1):e86998. doi: 10.1371/journal.pone.0086998 (PMC3906104; doi:10.1371/journal.pone.0086998)

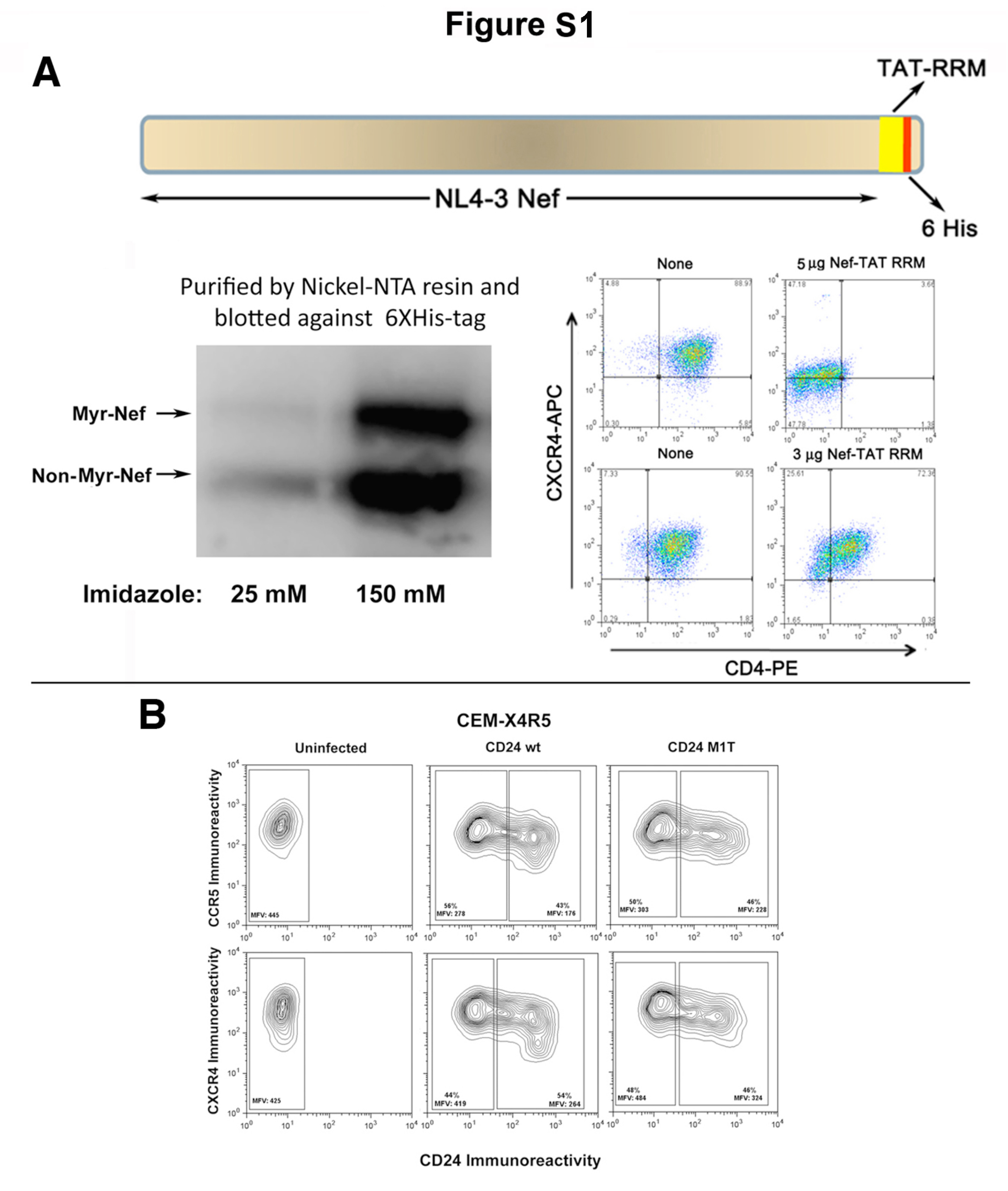

Supplement: Figure S1 — Recombinant Nef protein was taken up by lymphocytes and induced efficient downregulation of CD4 and CXCR4 in T lymphocytes. A) Schematic diagram (top) of recombinant Nef protein with the Tat RRM (arginine rich motif) domain appended at the C-terminus followed by 6 His residues, which was co-expressed in E. coli with the yeast N-myristoyl transferase. N-myristoyl or unmyristoylated Nef-Tat RRM protein(s) in E. coli extracts were purified by metal-affinity chromatography by successive batch elution with 25 and 100 nM imidazole. Proteins were resolved by SDS/PAGE, blotted and detected by chemiluminescence using Ni++-HRP (bottom left). Nef-Tat RRM induced marked downregulation of CD4 and CXCR4 in Jurkat cells. Jurkat cells (5×10^2^/ml) were incubated for the indicated times with affinity-purified Nef-Tat RRM (5 µg) in 0.2 ml of serum free RPMI prior immunological detection of CD4 and CXCR4 by flow cytometry. Bivariate FACS profiles of CXCR4 and CD4 are shown on the right. Results are representative on four experiments. B) Nef reduced the plasma membrane density of CCR5 and CXCR4 in a CEM cell line in the context of HIV infection CEM cells were infected with 400 ng p24 equivalent VSV-G pseudotyped NL4-3 HIV per 107 cells. HIVs used in this study express CD24 in place of VpR in a Nef (+) (CD24 wt) or a Nef (−) (CD24 M1T) background and have been described before [47]. Cells were harvested 24–36 h post infection. CD4, CCR5 or CXCR4 were detected using respective mAbs conjugated with APC.CD24 was detected using PE conjugated CD24 mAb. Bivariate FACS profiles of CCR5 or CXCR4 versus CD24 for uninfected, CD24 wt or CD24 M1T CEM cells with the respective receptor MFVs for the different gated populations are shown on the left. Results are representative of three experiments. (TIF) [file pone.0086998.s001.tif]

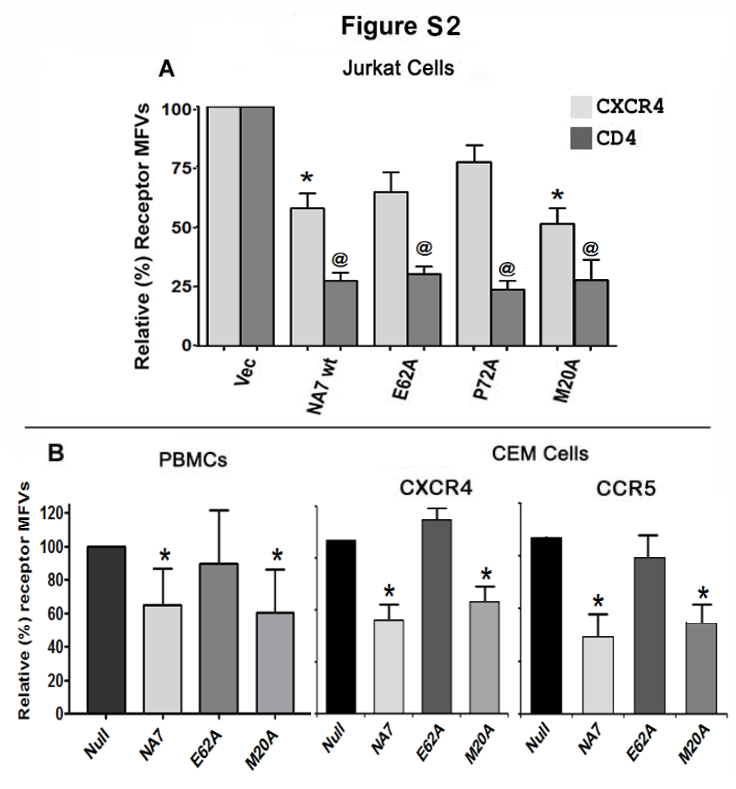

Supplement: Figure S2 — Nef induced CXCR4 downregulation by Nef was critically dependent on the tetra-glutamate and the poly-proline motifs of Nef. Effect of wt and mutant Nefs on native CXCR4 and CD4 was evaluated in Jurkat, CEM cells or fresh PBMCs (A and B). Cells were cotransfected with the indicated expression plasmids and a reference CD8 (Jurkat and CEM) or GFP (PBMCs) plasmid for gating. Histogram bars represent arithmetic means of MFVs, plotted with standard deviation (@n = 4, p<0.02; *n = 3, p<0.04). (TIF) [file pone.0086998.s002.tif]

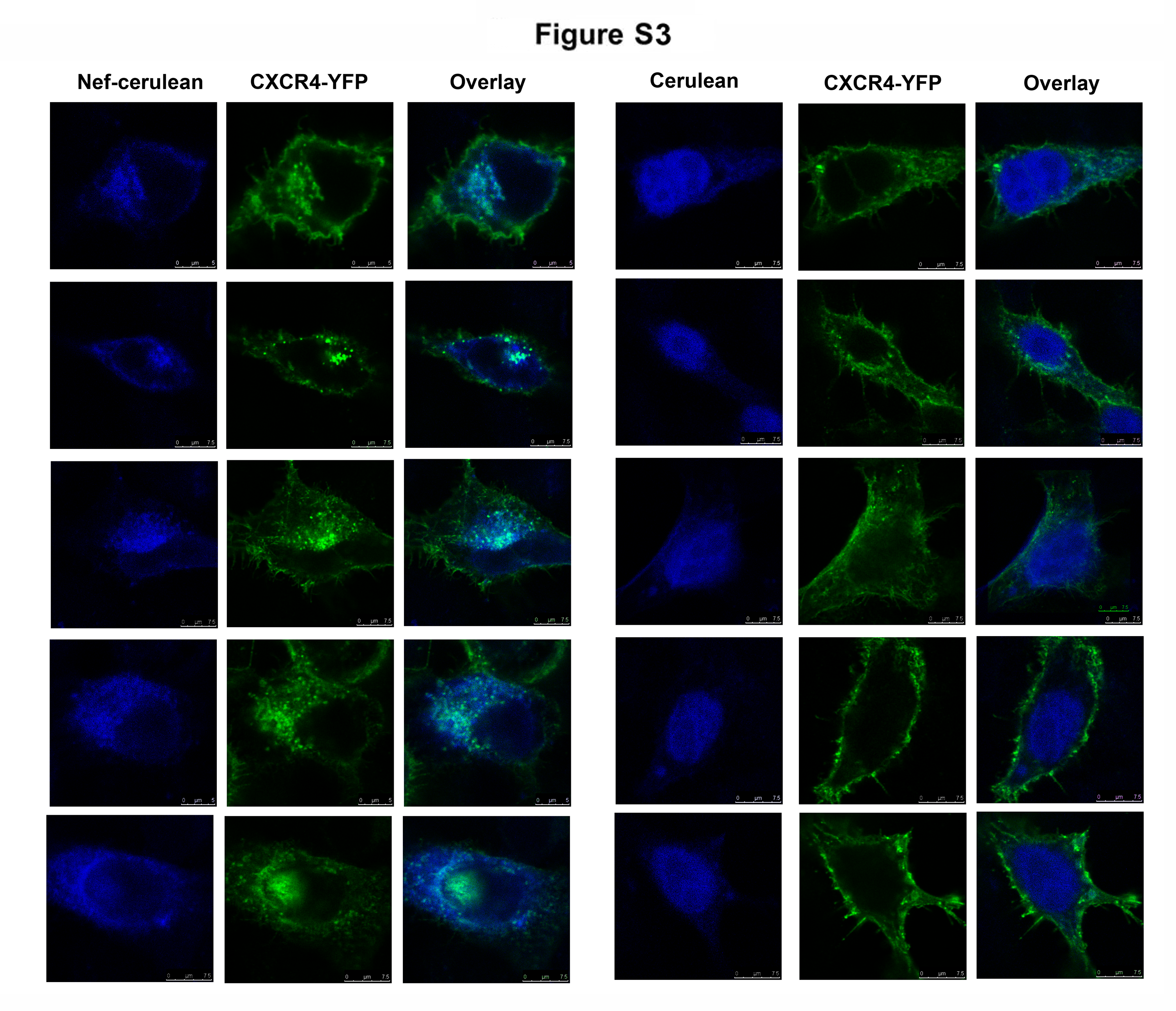

Supplement: Figure S3 — CXCR4 co-localizes with Nef in the perinuclear region: confocal microscopy was done on Hela cells transfected with CXCR4 YFP and Nef CerFP or Cer. Individual channels corresponding to CXCR4-YFP (G) and Nef-CerFP or Cer (B) fluorescence are shown alongside to the composite RGB images. Five representative fields with 7.5 or 10 µm scale bars are shown. (TIF) [file pone.0086998.s003.tif]
