# Supplementary material for: HIV-1 Nef Down-Modulates C-C and C-X-C Chemokine Receptors via Ubiquitin and Ubiquitin-Independent Mechanism
Source: PLoS One. 2014 Jan 29;9(1):e86998. doi: 10.1371/journal.pone.0086998 (PMC3906104; doi:10.1371/journal.pone.0086998)
Supplement: Procedures S1 — Bacterial expression of GST-Nef fusion protein. (DOCX) [file pone.0086998.s004.docx]

SUPPLEMENTAL EXPERIMENTAL PROCEDURES

Bacterial expression of GST-Nef fusion protein

The NL4-3 Nef containing TAT-RRM (arginine rich motif) was cloned into pET23d (EMD biosciences, Germany) and was co-expressed with plasmid encoding N-myristoyl transferase following the procedures as described [104]. The plasmid containing DNA of yeast N-myristoyl transferase (pBB131) was a gift from Jeff Gordon (Washington University, St.Louis, MO).

HIV infection

Pseudotyped HIV stocks of both Nef (+) HIV-1 NL4-3-r-HSA and Nef (-) NL4-3-M1T-HSA were prepared by transfecting 293T cells using TransIT LT1 transfection reagent (Mirus Bio LLC, WI) with 5 μg of HIV proviral DNA and 0.5 μg of VSV-G envelope DNA [47]. Virus in the supernatant was concentrated by ultracentrifugation using SW41 rotor (Beckman) at 35,000 rpm for 2 h at 4oC and resuspended in 1/20th original volume and was quantified by reverse transcriptase (RT) assay and ELISA for p24 antigen (PerkinElmer Life and Analytical Sciences, Netherlands). The viruses were adjusted for constant RT units/ml. Jurkat and CEM cells were infected in the presence of 4 μg/ml of polybrene (Millipore). After 3-5 days of infection, the cells were analyzed by multivariate quantitative flow cytometry and immuno-blotting cell lysates.
